# A method for deducing neck mobility in plesiosaurs, using the exceptionally preserved *Nichollssaura borealis*

**DOI:** 10.1098/rsos.172307

**Published:** 2018-08-01

**Authors:** Ramon S. Nagesan, Donald M. Henderson, Jason S. Anderson

**Affiliations:** 1 Department of Biological Sciences, University of Calgary, 2500 University Drive N.W., Calgary, Alberta, Canada T2N 1N4; 2 Royal Tyrrell Museum of Palaeontology, 1500 N Dinosaur Trail, Drumheller, Alberta, Canada T0J 0Y0; 3 Department of Comparative Biology and Experimental Medicine, Faculty of Veterinary Medicine, University of Calgary, 3330 Hospital Drive N.W., Calgary, Alberta, Canada T2N 4N1

**Keywords:** Plesiosauria, palaeontology, palaeoecology, neck mobility, range of motion, three-dimensional modelling

## Abstract

The elongate-necked aquatic plesiosaurs existed for 135 Myr during the Mesozoic. The function of this elongate neck is a point of debate. Using computed tomography and three-dimensional (3D) modelling, the range of motion (ROM) of the plesiosaur *Nichollssaura borealis* neck was assessed. To quantify the ROM, the intervertebral mobility was measured along the cervical vertebral column. This was done by manipulating the 3D models in the lateral and dorsoventral directions during two trials. The first assessed the mean intervertebral ROM between pairs of cervical vertebrae along the entire column, and the second assessed ROM with reduced intervertebral spaces. The results suggest that there may be preference for lateral neck movements in *N. borealis*, which could correspond to an ecological function related to prey capture. This study demonstrates that 3D modelling is an effective tool for assessing function morphology for structures where no good modern analogue exists.

## 1. Introduction

Plesiosauria is a clade of extinct, globally distributed marine reptiles that persisted for 135 Myr from the Triassic to the end Cretaceous [1–4]. Plesiosauria is rooted within the sauropterygian clade of reptiles, and may be a sister group to the lepidosaurs; however, sauroptergyian taxonomic relationships to other groups remain unclear [1]. Elongate necks, four paddle-like appendages and streamlined bodies exemplify the plesiosaur bauplan, which was adapted for a completely aquatic lifestyle [1,5,6]. There are three groups of uncertain relationship within Plesiosauria: the Plesiosauroidea, Pliosauridae and Rhomaleosauridae [4,7,8]. Plesiosauroidea comprised four subgroups: the Elasmosauridae, Leptocleidia, Cryptoclididae and Microcleididae [4]. This study focuses on a leptocleidid plesiosaur, *Nichollssaura borealis*.

The Plesiosauroidea generally have an elongate neck and a small skull, while Pliosauridae have a shorter neck and a larger robust skull [8]. The Plesiosauroidea have been hypothesized to be ambush predators, whereas members of the Pliosauridae are thought to have been pursuit predators [8]. However, new evidence suggests both morphological and ecological convergences may have occurred between the two groups [9,10].

The elongate necks of plesiosaurs evolved by the addition of cervical vertebrae; elasmosaurids are an extreme case with up to 76 cervical vertebrae and a 7 m long neck [11,12]. How the elongate neck functioned, what it functioned for and the ecological niches these predators occupied remain points of discussion [1–13]. Thus, understanding the range of motion (ROM) of the plesiosaur neck may help inform us how they were feeding by potentially defining the functional capabilities of the neck [14].

Zarnik [15], Welles [16], Evans [17] and Zammit *et al*. [18] previously quantified the function of the plesiosaur neck by inferring the ROM along the cervical vertebral column. Zarnik [15] and Evans [17] used morphological measurements to determine how far each cervical vertebra could move in relation to another. Zarnik [15] and Evans [17] measured the zygapophyseal angles, neural spine height and centrum dimensions on plesiosaur cervical vertebrae to inform their ROM interpretations. Evans [17] calculated the possible ROM of the necks of two plesiosaurs: *Cryptoclidus eurymerus* and *Muraenosaurus leedsii*. Evans's [17] calculations relied on assumptions based on the relative position of the zygapophyses during movement and took into account an estimation of intervertebral spacing. However, both Zarnik [15] and Evans [17] did not conduct any articulations because the cervical vertebral columns from their specimens were incomplete, damaged or distorted. Zammit *et al*. [18], like Welles [16], created two-dimensional (2D) models based on measurements of several nearly complete elasmosaurid plesiosaurs. The models were constructed out of cardboard in dorsal and lateral views [18]. According to their 2D model, the mean intervertebral flexibility decreased posteriorly in the column by 6° dorsally, 2° ventrally and 4° laterally (trends across the various specimens) [18]. Zammit *et al*. [18] suggested that greater range of neck flexibility in the ventral and lateral directions compared with the dorsal direction may be common among plesiosaurs, while Evans [17] found that the lateral ROM was greater than the dorsoventral. The difference between the ROM patterns found in these two studies [17,18] suggests that plesiosaur neck function, and the resulting ecologies, may have varied across the group.

These previous studies [15–18] were based on approximations or 2D analogues due to the limits of the available materials. Their conclusions may be refined by using precise three-dimensional (3D) modelling techniques that are based on the preserved morphology. The vertebral functions of stem tetrapods, turtles and crocodilians have been previously analysed using 3D models produced from computed tomography (CT) scans [19–21]. In the case of turtles and crocodilians, these 3D models have been combined with morphological studies to assess the ROM in the vertebral column [20,21]. CT scans are compiled into 3D models that are manipulated on a computer to simulate the ROM of the vertebral column [22]. Werneburg *et al*. [20] assessed the ROM in turtle necks to understand the evolution of neck retraction into their shells. Those models were paired with fossil specimens to look at how the articular facets, such as the zygapophyses, allow for extreme ROM to pull the neck into the shell in an ‘S’ curve [20]. Molnar *et al*. [21] calculated crocodilian vertebral column flexibility with 3D models built from CT scans of both extinct and extant specimens. Crocodilian trunk flexibility was then tied to evolutionary changes in the hypothesized habitats that both extant and extinct crocodilians may have lived in [21]. In both cases, the 3D models allowed for very precise measurements of vertebral column ROM.

The purpose of this study is to assess plesiosaur cervical vertebral column ROM. With a well-persevered and fully articulated specimen, it is possible to generate 3D models that can be manipulated to assess the intervertebral mobility along the cervical vertebral column. The increased precision of 3D modelling may allow for a clearer picture of how the plesiosaur, used in this study, was able to move its neck and provide a methodology for studying neck movements in the rest of the group. A more complete picture of plesiosaur neck mobility will also aid in our understanding of the ecological niches that this group may have filled.

## 2. Material and methods

### 2.1. Institutional abbreviations

TMP: Royal Tyrrell Museum of Palaeontology, Drumheller, Canada.

### 2.2. *Nichollssaura borealis* (TMP 1994.122.0001)

**Figure 1.**
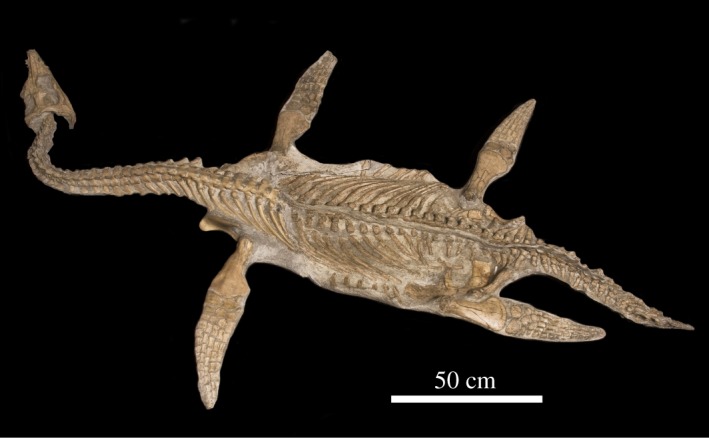
The entire skeleton of *Nichollssaura borealis* (TMP 1994.122.0001) in dorsal view. The specimen is accessioned at the Royal Tyrrell Museum of Palaeontology. Total body length is 260 cm. Image courtesy of Royal Tyrrell Museum of Paleontology.

**Figure 2.**
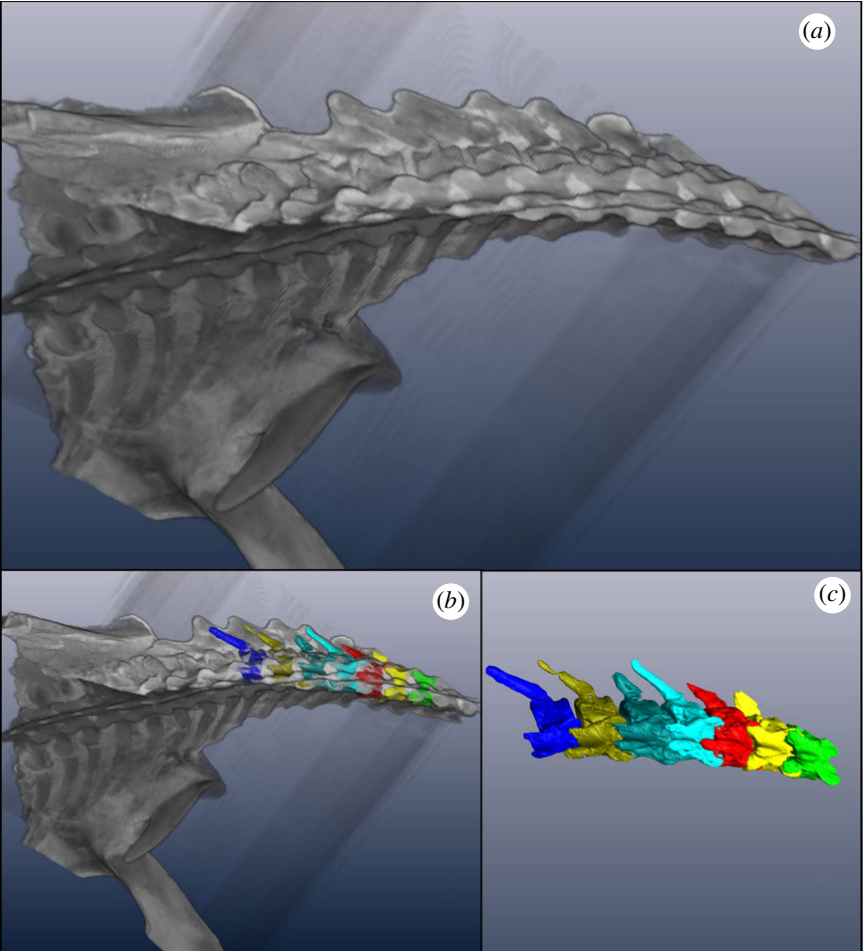
(*a*) Isosurface of *Nichollssaura borealis* produced in Amira 5.4 from CT scans, in dorsal view (dv). (*b*) Volumized cervical vertebrae (dv). (*c*) Isolated cervical vertebrae (dv), with isosurface removed. This process continues until all the cervical vertebrae are isolated out by volumization in Amira 5.4.

An exemplar plesiosaur, *N. borealis* [23,24] (TMP 1994.122.0001, figure 1), was selected to construct 3D models (figure 2) because of its availability and capability of fitting into a medical CT scanner. It is the holotype and only specimen of its species [23]. *N. borealis* was exceptionally preserved and fully articulated, and it was 260 cm long [23]. The specimen was excavated in 1994 from the Wabiska Member of the Clearwater formation (Albian, 113 Ma) near Fort McMurray, Alberta, Canada [23,24]. The skull and cervical vertebral column were present, with some damage caused to a few cervical vertebrae during excavation (i.e. lost neural spines, missing cervical ribs and severed centra). According to Druckenmiller & Russell [23,24], there are 24 cervical vertebrae and this study followed that count. There was a minimal amount of lateral deformation through the frontal plane of *N. borealis* that can be observed in the CT scans. This minimal deformation was not corrected for in this study because of the uncertainty that may be introduced by reconstructing the morphology. There does not appear to be any deformation from compressive forces in any plane. Because of this and the complete articulation of the cervical vertebral column, we assume that the length of the vertebral column and intervertebral space approximates the life-like condition of *N. borealis*' neck [21].

### 2.3. Computed tomography

CT scanning of *N. borealis* (figure 1) [23,24] was carried out to create 3D renders of the cervical vertebral column for a biomechanical model by R. Myszkowski in Calgary, Alberta, at Mayfair Diagnostics on a General Electric HD 705 64-slice CT scanner. The skull and vertebral column were scanned at 120 kV, 185 µA and 0.625 mm slice thickness with a 512 × 512 matrix, resulting in a voxel size of 0.625 mm × 0.625 mm × 1.25 mm (a 512 matrix was the scan resolution of the General Electric HD 705 64-slice CT scanner). We downsampled the scans of *N. borealis* in ImageJ [25] from 1400 to 700 slices by removing every other slice, and cropped to remove non-informative image data. We organized the slices into an image sequence using a virtual stack (data are accessible via Dryad).

### 2.4. Three-dimensional model rendering

The 3D visualization package Amira 5.4 (FEI, Oregon) allowed us to render the CT scan image sequences into exportable 3D models (figure 2). In order to generate a model of the morphology of interest, in this case the cervical vertebral column, we created a ‘label-field’ using the label-field module. Each cervical vertebra was segmented into a 3D model, referred to as a ‘material’ in Amira 5.4. This was repeated until the entire cervical vertebral column was segmented into 3D models.

### 2.5. Model manipulations

The primary ROM data collection was conducted in Autodesk Maya (Autodesk, 2015). Autodesk Maya's interface may be used to create 3D animations, or, in this case, ROM profiles.

To export the cervical vertebrae from Amira 5.4 to Autodesk Maya, each cervical vertebra was isolated from the rest of the vertebral column as its own file. This allowed the cervical vertebrae to be manipulated against each other, and the ROM to be measured. With the ‘arithmetic module’ in Amira 5.4, a specific material in the cervical vertebral column label-field module was selected and re-segmented. From the re-segmented CT scans, a new surface was produced with the ‘surface gen module’, and the resulting model was saved as an object file (.obj). This was repeated for each of the materials associated with the cervical vertebrae.

In Autodesk Maya, a ‘new project’ was created into which the .obj files were imported. For the first and second trials (see §§2.6 and 2.7), only two .obj files were imported at any one time, and at no point was an entire cervical column on screen. For the third trial (see §2.9), all the .obj files were imported (one at a time) to produce an entire cervical series that was then manipulated.

**Figure 3.**
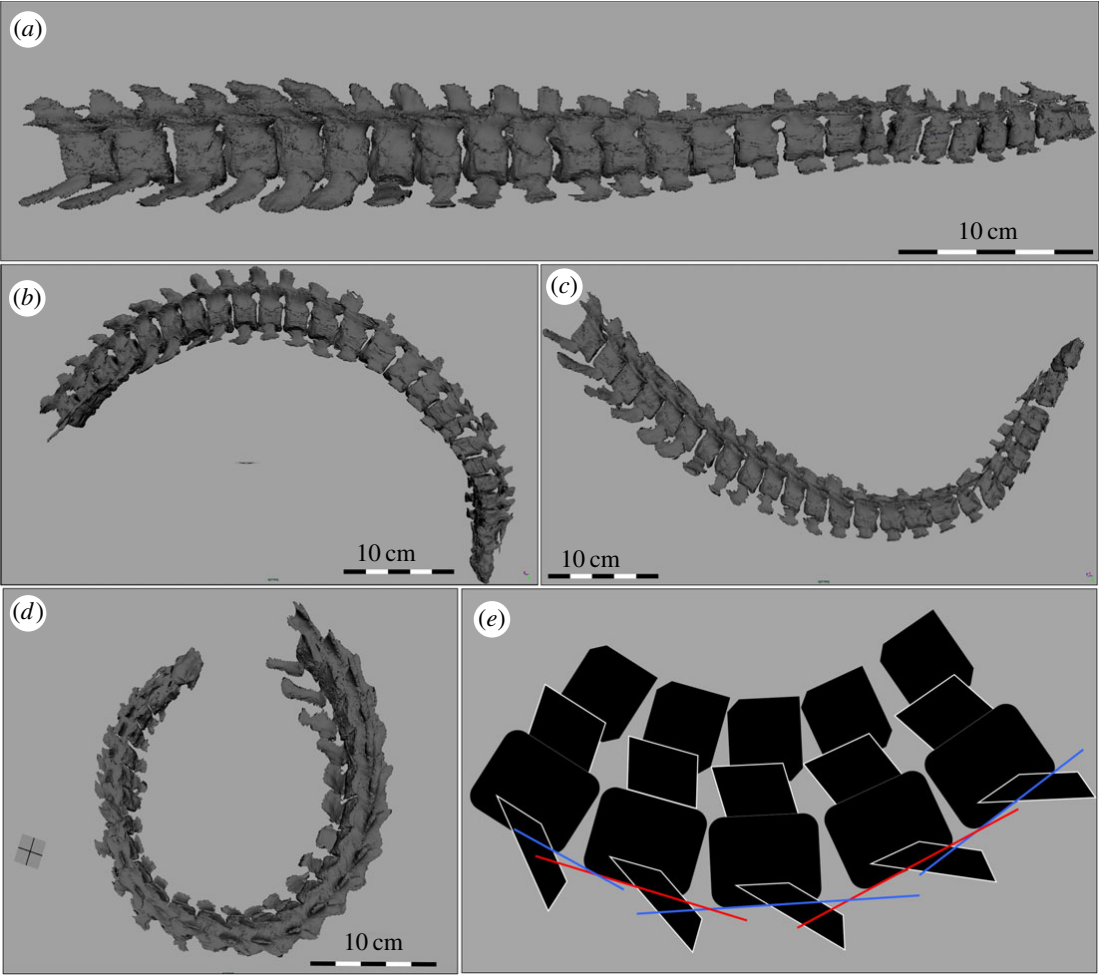
A visualization of the range of motion profiles for the complete neck of *Nichollssaura borealis.* (*a*) Left lateral view (llv) of the neutral starting position of the rearticulated neck of *N. borealis*; (*b*) maximum ventral flexion (llv); (*c*) maximum dorsal flexion (llv); (*d*) maximum lateral flexion in dorsal view; and (*e*) schematic of flexed vertebra (llv).

**Figure 4.**
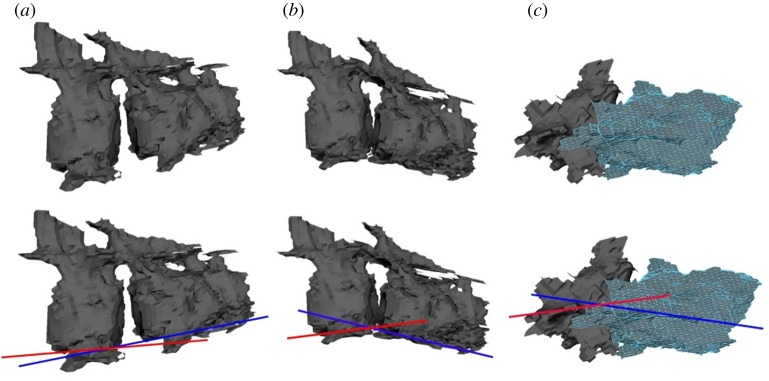
Paired cervical vertebral mobility (PCVM) trial: atlas-axis articulation with C3 in *N. borealis*. (*a*) Dorsal mobility profile (mp) and measurement right lateral view (rlv.). (*b*) Ventral mp and measurement rlv. (*c*) Lateral mp and measurement dorsal view.

A lateral, dorsal and ventral ROM profile was created for each manipulation trial (figure 3 shows a visualization of the ROM profiles for the complete neck). The first trial assessed mobility between paired cervical vertebrae (PCVM) (figure 4), and the second trial assessed the minimum intervertebral space mobility (MISM). The lateral mobility profile corresponded to movement in the frontal plane. The dorsal and ventral mobility profiles corresponded to flexion and extension in the median plane. Each cervical vertebra was manipulated with respect to the vertebra anterior to it in the column. For all manipulations, the point of rotation was placed between the centra, specifically at the central point of the intervertebral discs. This assumption follows previous neck mobility studies [15,16,18].

The manipulations of the cervical vertebrae were conducted until maximum displacement, defined as when bone-on-bone contact between vertebra was reached [21,26]. The 3D models were set to ‘rigid bodies’ in Autodesk Maya, which prevented them from passing through one another. Bone-on-bone contact occurred between the zygapophyses, centra, neural spines or cervical ribs, respectively, depending on the direction of movement. Bone-on-bone contact would not take place in a living animal because of the soft tissue structures encasing the bone. As a result these manipulations represent the osteological maximums of movement in the frontal and median planes. Bone-on-bone contact was assumed as the point of maximum displacement to avoid estimation error associated with reconstructing the thickness of the soft tissues (see Discussion and conclusion).

### 2.6. Paired cervical vertebral mobility trial

In this trial, the PCVM was assessed along the entire cervical vertebral column. To do this, the two cervical vertebrae were uploaded as .obj files into the same 3D ‘scene’ in Autodesk Maya. The two cervical vertebrae were first manipulated into a ‘neutral’ starting position; this was not the osteological neutral pose proposed by Stevens & Parrish [14] because others have argued that it does not realistically or accurately reflect a resting position of a neck [27,28]. Instead, the neutral starting position in this analysis was a straightened neck using the preserved intervertebral space (figure 3*a*), with the assumption that the preserved space remains close to what was present in life given the overall quality of, and lack of compression to, the specimen [21,29]. From the neutral starting position the cervical vertebrae were manipulated into the lateral, dorsal and ventral mobility profiles (figures 3 and 4).

By manipulating two cervical vertebrae at a time, rather than the entire vertebral column, the 3D tools were easier to manage in Autodesk Maya. This streamlined the manipulation process and may have reduced user error during manipulation. When manipulating an entire vertebral column in Autodesk Maya, both user and observational errors may have resulted from having all of the different models on screen at one time.

Starting from the atlas-axis and C3, each pair of vertebrae would be uploaded such that only two were on screen at a time. Once the manipulation between two vertebrae was complete, the anterior vertebra in the pair was removed and the next vertebra in the column was uploaded. For example, following the manipulation of the atlas-axis with C3, the atlas-axis was removed leaving C3 on screen and C4 was uploaded for a manipulation with C3. After each manipulation was completed, a screen capture was taken for ROM measurements. This continued in sequence until the entire vertebral column had been manipulated into the three mobility profiles (lateral, dorsal and ventral).

### 2.7. Minimum intervertebral space mobility trial

To assess other possibilities for the ROM with different intervertebral spacing, we conducted a second set of manipulations (see electronic supplementary material, figure S.1). The intervertebral spacing was reduced until the centra of the cervical vertebrae were in contact with one another. As a result, contact at the centra could not be used to define the end of manipulation (PCVM trials). Instead, the maximum displacement was assessed when there was contact at any of the other possible points of contact (see above). Although no compression to the vertebral column was evident, it was possible that taphonomic and diagenetic processes have altered the original intervertebral spaces at the millimetre scale. Evans [17] and Zammit *et al*. [18] showed that increased intervertebral spaces correlated to increased neck ROM. This second trial allowed us to bracket the possible ROM of the neck of *N. borealis* between zero intervertebral space and what was preserved (PCVM trials). From here the cervical vertebrae were manipulated into the same three mobility profiles as in the previous trial. As per the method of the PCVM trial, the vertebrae were manipulated two at a time, with one vertebra being manipulated with respect to another in the dorsal, ventral and lateral directions.

### 2.8. Varanus dumerilii

An extant varanid, *Varanus dumerilii* [30], was used to assess if the 3D models of *N. borealis* present a realistic interpretation for the mobility of the neck. *V. dumerilii* was chosen because of its relatively long neck among lepidosaurs and its availability. This specimen was fixed in 10% buffered formalin, and then transferred to storage in 70% ethanol for use in another study in 2012. The specimen measured approximately 26 cm long and was a juvenile. The specimen was micro-CT-scanned, 3D modelled and radiographed (a step not possible for *N. borealis*) to approximate the ROM with soft tissues taken into account. The varanid was modelled using the same methods applied to *N. borealis*. The varanid offered the ability to also model ROM with radiographs to assess the difference in the ROM when musculature was also considered. By having both metrics, we can assess whether the 3D computer models (which lack musculature) match the ROM of an animal with musculature. If those two measurements were not significantly different, then we reasoned that the plesiosaur model presented a valid approximation for the ROM should the musculature also have been present.

### 2.9. *Varanus dumerilii* 3D model trial

**Figure 5.**
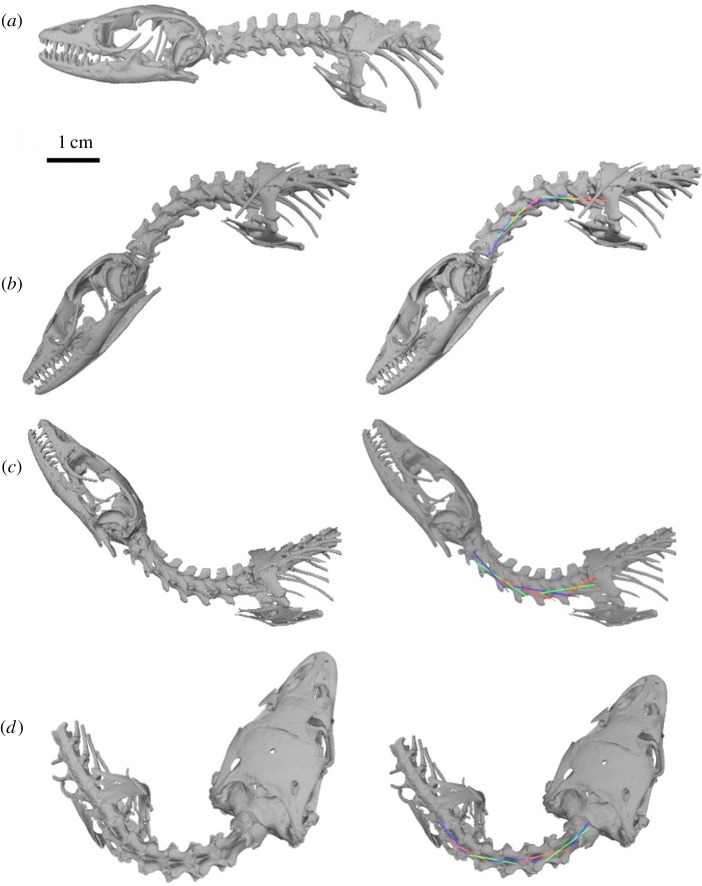
*Varanus dumerilii* 3D model (V3DM) trial: articulations of *Varanus dumerilii* in Autodesk Maya. (*a*) Left lateral view (llv) of straightened neutral starting position; (*b*) ventral articulation (llv); (*c*) dorsal articulation (llv); (*d*) lateral articulation in dorsal view.

As in the ROM manipulations conducted for *N. borealis*, the cervical vertebral column of *Varanus dumerilii* was straightened into the neutral starting position (figure 5*a*). The point of rotation was placed at the intervertebral spaces, as in *N. borealis*, which follows previous studies [17,18,20,21]. From the neutral starting position, the neck was manipulated into dorsal, ventral and lateral mobility profiles until maximum displacement (figure 5*b–d*).

### 2.10. Radiograph mobility of *Varanus dumerilii* trial

**Figure 6.**
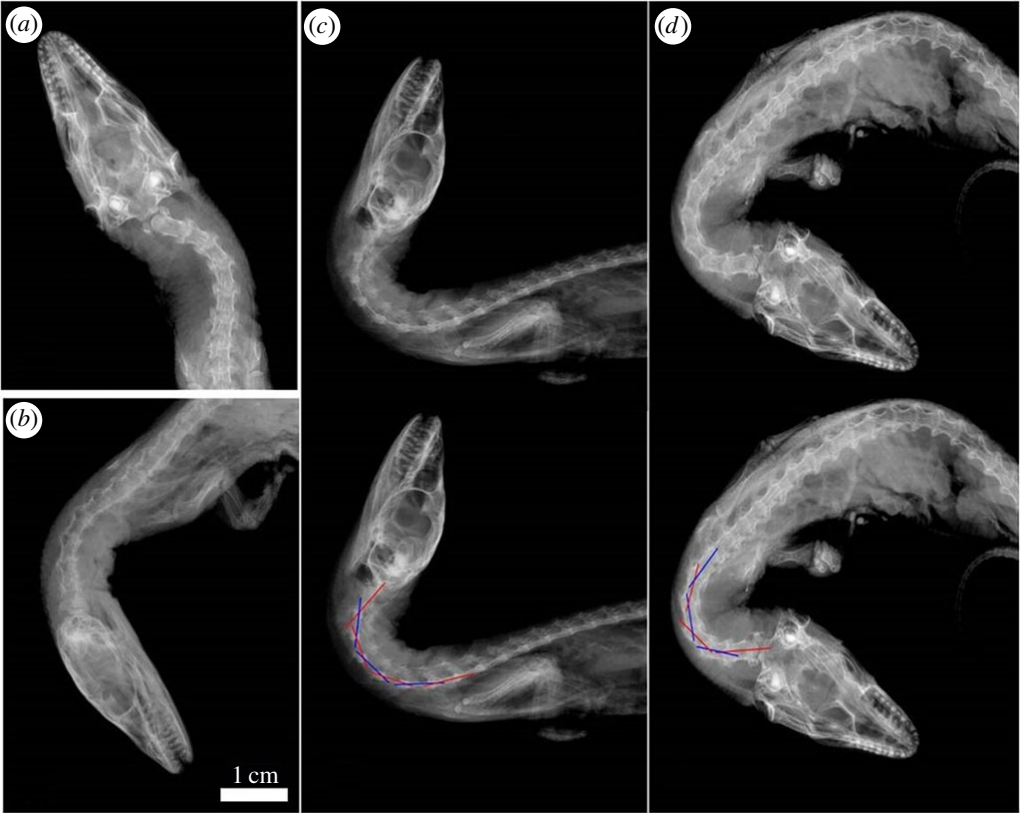
Radiographed model of *Varanus dumerilii* (RMV trial): radiographs showing the various articulations of *Varanus dumerilii*. (*a*) An unaltered radiograph of the specimen, the specimen sits as it was preserved. (*b*) Ventral articulation, in left lateral view. (*c*) Dorsal articulation in left lateral view: above, without lines for angle measurements; below, with lines for angle measurements. (*d*) Lateral articulation in dorsal view: above, without lines for angle measurements; below, with lines for angle measurements.

To assess the maximum ROM of an elongate reptilian neck with soft tissues, a series of radiographs were taken of the specimen of *Varanus dumerilii* on a Kubtec Xpert 80-L flatbed radiography system at the University of Calgary, Calgary, Alberta (figure 6) [31]. The specimen was placed on the flatbed and positioned directly under the emitter. Adhesive tape was used to hold the neck in the various positions because it was radio-transparent (A.P. Russell 2016, personal communication). The ROM manipulations for *V. dumerilii* follow the methodology laid out by Werneburg *et al*. [20].

To assess the neck's maximum ROM, three sets of radiographs were produced. First, the neck was held in the neutral starting position—straight without flexion in any of the three directions. For the neutral starting position of the dorsal and ventral ROM profiles, *V. dumerilii* was placed in left lateral recumbence. To assess dorsal ROM, the neck was elevated (raising the head) to the point at which it could no longer mechanically be moved without excessive force, and was held in place with adhesive tape. Similarly, the neck was depressed for the ventral ROM. To assess the lateral ROM, the specimen was placed ventral side down on the radiograph platform. The neck was then bent by hand to the right, to the point where it could not mechanically be moved without excessive force, and held in place with adhesive tape. After each radiograph was taken, the profile was changed.

A total of 32 radiographs were taken at 30 kV and 910 µA, of which approximately 12 were clear enough for data collection (figure 6). The radiographs were captured using the Kubtec Xpert imaging software and were saved in dicom format. The radiographs were converted from dicom to .jpeg format by using the export function in the Kubtec Xpert 80 l imaging software. The radiographs were then exported for data collection.

### 2.11. Angle measurements of *Nichollssaura borealis*

Screen captures of each of the ROM profiles were imported into Adobe Illustrator (AI). For the dorsal and ventral mobility profiles, a straight line was drawn along the base of the centrum of each of the vertebrae (see electronic supplementary material, figure S.2a). The line was extended out beyond the length of the vertebra on which it was drawn; as a result, a series of intersecting lines was created for each ROM profile (see electronic supplementary material, figure S.2b). For the lateral ROM profile, the straight lines were placed along the median plane in dorsal view of each vertebra (see electronic supplementary material, figure S.2c), and, again, extended beyond the length of the vertebra on which it was placed, resulting in a series of intersecting lines.

Once the lines were fitted each screen capture was uploaded to ImageJ. Using the measurement tool in ImageJ, the angle between the intersecting lines (see electronic supplementary material, figure S.2) for each pair of cervical vertebrae was recorded. Each angle (see electronic supplementary material, figure S.2) was measured three times and their means were taken as the ROM between two cervical vertebrae (raw data with standard deviation and uncertainty are accessible via the electronic supplementary material).

### 2.12. Angle measurements of *Varanus dumerilii* 3D model

The data collection from *Varanus dumerilii* followed closely that of *N. borealis*, differing only in where the lines were placed. The base of the centra was not used because of the nonlinear shape of the hypapophysis on the cervical vertebra (see electronic supplementary material, figure S.3). Instead, the lines were drawn through the lateral keel of the cervical centra (see electronic supplementary material, figure S.3). This position gives equivalent results to measuring along the base of the centrum; the only difference was that the lines were moved upwards. Each line was extended out beyond the vertebra on which it was drawn, resulting in a series of intersecting lines. The lateral angle measurements follow the same method as for *N. borealis*. The angles of intersection were measured in ImageJ (see electronic supplementary material, figure S.3; also see the electronic supplementary material for the raw data).

### 2.13. Angle measurements of radiographed mobility of *Varanus dumerilii*

The radiographs were converted to .jpeg format and uploaded into AI. In AI, lines were fitted following the lateral keel of the cervical centra, similar to the lines fitted for the Autodesk Maya 3D model (above) of *Varanus dumerilii* (figure 5; electronic supplementary material, figure S.3). The lateral angle measurements follow the same method for *N. borealis*. The angles of intersection were measured three times in ImageJ (see the electronic supplementary material for the raw data).

### 2.14. Statistical analysis

The angular measurements could not be analysed using the same tests that would be used for linear measurements (i.e. morphological dimensions or count data). To assess whether there were differences between the ROM profiles circular statistics were used. Circular (also called directional) statistical tests analyse datasets where each datum was measured as an angle from a point on a circle. To conduct the analyses, the angular measurements were converted to radians. In this case, each circular datum was an angle of movement between two cervical vertebrae.

The Watson–Williams test for equal means was used to assess if the mean intervertebral ROM in one direction differs from that of another. The null hypothesis was that the mean intervertebral ROM in any direction of movement was not different from any other.

First, the ROM profiles of *N. borealis* were compared with one another (i.e. PCVM: lateral mobility versus dorsal mobility; PCVM: lateral mobility versus ventral mobility; and PCVM: dorsal mobility versus ventral mobility). Second, *N. borealis* mobility profiles were compared between trials (i.e. PCVM: lateral mobility versus MISM: lateral mobility).

To assess if the 3D models approximate the ROM of a neck with soft tissues Watson–Williams tests were conducted between the varanid Autodesk Maya V3DM trial and the varanid radiograph RMV trial ROM (i.e. V3DM lateral mobility profile versus RMV lateral mobility profile; V3DM dorsal mobility profile versus RMV dorsal mobility profiles). All statistical tests were conducted with PAST v. 3 [32].

## 3. Results

**Figure 7.**
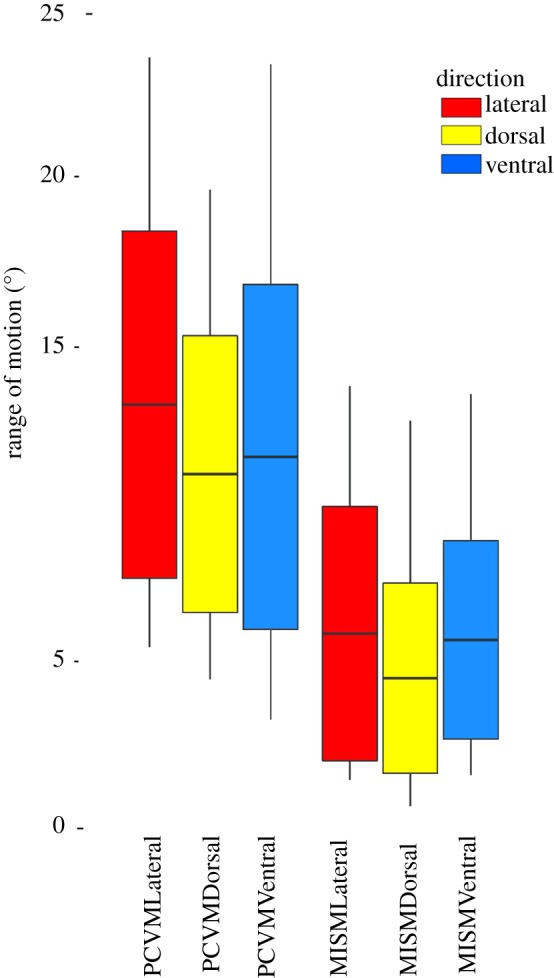
The range of motion (in degrees) for the neck of *Nichollssaura borealis* is shown as a boxplot with the mean and standard deviation shown. Each of the two trials is plotted with each of its range of motion profiles: lateral, dorsal and ventral.

**Figure 8.**
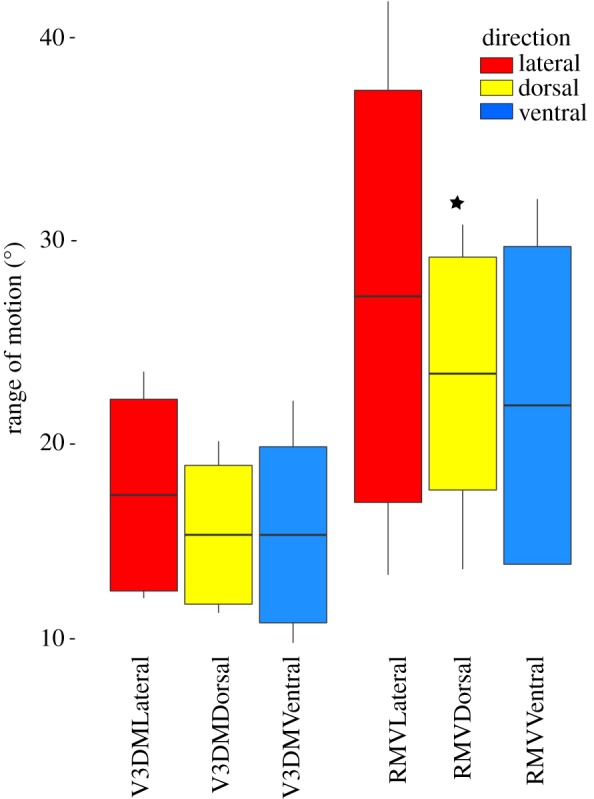
The range of motion (in degrees) for the neck of *Varanus dumerilii* is shown as a boxplot with the mean and standard deviation shown. Each of the two trials is plotted with each of its range of motion profiles: lateral, dorsal and ventral. The star indicates significance (table 4).

The results of the mobility profile measurements can be found in tables 1 and 2 (data are accessible via Dryad) (figures 7 and 8). A summary of results of the statistical analyses can be found in tables 3 and 4. In this section, only the significant results are presented.

**Table 1.** Results of the paired cervical vertebral mobility (PCVM) and minimum intervertebral space mobility (MISM) trials mobility profiles of *Nichollssaura borealis*. Each trial shows the results for the lateral (Lat.), dorsal (Dors.) and ventral (Vent.) mobility profiles, with the mean representing the mean intervertebral range of motion. Standard deviation is given below the mean, and total mobility of the neck is shown as the sum of the mobility of each pair of cervical vertebrae in the neck.

	trial					
	PCVM			MISM		
	Lat.	Dors.	Vent.	Lat.	Dors.	Vent.
*n*	20	20	20	20	18	19
mean	12.92	10.786	11.316	5.29	4.5	5.38
s.d.	5.20	4.15	5.17	3.80	2.84	2.97
min.	5.46	4.47	3.23	0	0.571	0
max.	23.61	19.54	23.38	13.48	12.43	13.25
sum	258.47	215.72	226.32	105.81	81.14	102.29

**Table 2.** Results of the *Varanus dumerilii* 3D model (V3DM) and radiographed mobility of *Varanus dumerilii* (RMV) trials mobility profiles of *Varanus dumerilii*. Each trial shows the results for the lateral (Lat.), dorsal (Dors.) and ventral (Vent.) mobility profiles, with the mean representing the mean intervertebral range of motion. Standard deviation is given below the mean, and total mobility of the neck is shown as the sum of the mobility of each pair of cervical vertebrae in the neck.

	trial					
	V3DM			RMV		
	Lat.	Dors.	Vent.	Lat.	Dors.	Vent.
*n*	6	6	5	5	6	5
mean	17.34	15.38	15.38	27.156	23.34	21.77
s.d.	4.32	3.07	3.89	9.10	5.25	7.02
min.	12.25	11.52	10.04	13.4	13.68	13.96
max.	23.45	20.01	22.003	41.74	30.70	31.97
sum	104.05	92.28	76.9	135.78	140.05	108.87

**Table 3.** A summary of the Watson–Williams tests for equal means, between the different range of motion profiles (mp). Paired cervical vertebral mobility (PCVM): lateral mp (PCVMLat), PCVM: doral mp (PCVMDors), PCVM: ventral mp (PCVMVent); minimum intervertebral space mobility (MISM): lateral mp (MISMLat), MISM: dorsal mp (MISMDors) and MISM: ventral mp (MISMVent).

	PCVMDors	PCVMVent	MISMLat	MISMDors	MISMVent
PCVMLat	0.16952	0.34534	1.0898 × 10^−5^*		
PCVMDors		0.72922		7.1582 × 10^−6^*	
PCVMVent					0.00016659*
MISMLat				0.50815	0.93784
MISMDors					0.39463

*p ≤ 0.05.

### 3.1. Significant results of the *Nichollssaura borealis* Watson–Williams comparisons

The mean intervertebral ROM in the lateral and dorsal mobility profiles of the PCVM trial was significantly greater than that in the MISM trial (table 3). The mean intervertebral ROM in the ventral mobility profile of the PCVM trial was significantly greater than that in the MISM trial (table 3).

### 3.2. Significant results for *Varanus dumerilii* comparisons

The mean intervertebral ROM in the dorsal direction is significantly greater for the radiographs than for the Autodesk Maya model (table 4 and figure 8).

**Table 4.** A summary of the Watson–Williams tests for equal means, between the different range of motion profiles for *Varanus dumerilii*. *V. dumerilii* 3D model (V3DM): lateral mobility profile (mp) (V3DMLat), dorsal mp (V3DMDors), ventral mp (V3DMVent); radiographed mobility of *Varanus dumerilii* (RMV): lateral mp (RMVLat), dorsal mp (RMVDors), ventral mp (RMVVent).

	RMVLat	RMVDors	RMVVent
V3DMLat	0.063135		
V3DMDors		0.013548*	
V3DMVent			0.94331

*p ≤ 0.05.

## 4. Discussion and conclusion

### 4.1. Range of motion

The mean intervertebral mobility from the PCVM trial represents the osteological maximum ROM for the neck. In the PCVM trial, the mean intervertebral ROM was not significantly different between the lateral, dorsal or ventral profiles (table 3); however, the mean intervertebral mobility of the lateral plane was highest at 12.92° (*s* = 5.20°) (figure 7). This finding may indicate that the lateral mobility profile was the preferred plane of neck movement in *N. borealis*.

In the MISM trial, we reduced the intervertebral space before conducting the ROM manipulations in Autodesk Maya. This simulated neck movement if the cervical vertebrae were locked together in a much closer position than was preserved, and established the osteological minimum ROM. We expected minimal to no movement between the vertebrae, but found that some still occurred. The mean intervertebral mobility did not differ between the three profiles and was significantly less than the equivalent mobility profile in the PCVM trial. This was in line with what we expected to see when the intervertebral space between two cervical vertebrae was reduced, or removed [15].

Given our assumptions, the neck mobility profiles presented in this study represent the osteological maximal (PCVM trials) and minimal (MISM trial) ROM of *N. borealis* neck. There may be a preference for neck movement in the lateral direction, up to a maximum mean intervertebral mobility of 12.92° (*s* = 5.20°) (PCVM) and a minimum mobility of 5.29° (*s* = 3.8°) (MISM) (figure 7). The mean intervertebral mobility in the dorsal direction ranged from a maximum of 10.79° (*s* = 4.15°) (PCVM) to a minimum of 4.50° (*s* = 2.84°) (MISM), and in the ventral plane from a maximum of 11.51° (*s* = 5.17°) (PCVM) to a minimum of 5.38° (*s* = 2.97°) (MISM) (figure 7). The lateral mobility profile shows the greatest mean intervertebral mobility, and greatest range between minimum and maximum mobility.

We chose not to conduct a set of manipulations with increased intervertebral spacing over that which was preserved in *N. borealis*. Although it may be possible that the soft tissue structure shrank during preservation, we found no robust way of estimating realistic increases to the intervertebral space in the literature. As no compression was observed to have occurred to *N. borealis*, it was reasonable to assume that the preserved spacing closely approximates the life-like condition [21]. Additionally with the preserved intervertebral spacing the zygapophyses appear to overlap naturally, reflecting a state we would expect to see in a living animal. This observation lends support to our determination that the preserved intervertebral spacing does approximate the spacing that was present during *N. boreali*s' life.

To assess if the 3D models of *N. borealis* represent a biologically realistic system for estimating neck mobility, the extant varanid ROM study was conducted. First, a 3D model of *V. dumerilii* was produced from CT scans and then it was manipulated into the three mobility profiles in Autodesk Maya. Then, a series of radiographs were taken of manipulations of the same three mobility profiles of the *V. dumerilii*. A comparison of the two datasets allowed for a direct comparison between mobility models both with and without soft tissue contributions. There was no significant difference between the mean intervertebral mobility in either the lateral or ventral plane between the 3D model and the radiographed manipulations. However, the 3D model (V3DM) appeared to have significantly underestimated the mean intervertebral dorsal mobility compared with the radiographs (RMV) ((15.38° (*s* = 3.07°) versus 23.34° (*s* = 5.25°)) (figure 8). This underestimation may be caused by the soft tissue morphology in *V. dumerilii* that allows for more dorso-extension; however, it is difficult to be certain without further soft tissue analysis. Cobley *et al*. [33] found that presence of soft tissue can actually increase intervertebral ROM. The difference in the dorsal mobility may also be caused by the fact that, when radiographed, the specimen had been preserved for some time. Prior to preservation in formalin, the specimen had been frozen and thawed, which probably resulted in some tissue degeneration. This degeneration and the long period of storage could have resulted in non-natural movement patterns in the manipulated neck. It is also possible that the difference may be due to the limitations that result from trying to simulate complex vertebral movements with 3D models. For example, in the dorsal direction, there may also be some translational movement between the vertebrae, a pattern which was not captured by the models. Modelling of translational and rotational movement between cervical vertebrae should be conducted in the future. However, the overall congruence between the 3D model and the radiograph manipulations of *V. dumerilii* suggests that this type of biomechanical manipulation can accurately estimate neck mobility in a biologically realistic manner for a plesiosaur such as *N. borealis*.

### 4.2. Constraints on neck mobility

During the 3D manipulations of *N. borealis*, we found several constraints on neck mobility. Since the neural spine, zygapophyses, centra and cervical ribs marked points of bone-on-bone contact (maximum displacement), then they also served as the main osteological constraints to movement of the neck. In the lateral mobility profile, the primary osteological constraints were the cervical ribs because they project laterally and came into contact during medio-lateral movement. In the dorsal mobility profile, the point of bone-on-bone contact was between the pre- and postzygapophyses. Changes in the angulation of the zygapophyses can also affect the ROM of the neck [17]. In the posterior cervical vertebrae, bone-on-bone contact between subsequent neural spines constrained ROM to a greater degree than the zygapophyses. The change in the point of bone-on-bone contact is likely to be associated with the change in the morphology of the cervical vertebrae. Further back along the cervical vertebral column the neural spines became more posteriorly angled and increased in height. In the ventral mobility profile, the anterior cervical vertebrae were constrained by the ventral aspects of the ‘faces’ of each centrum.

While it is important to determine the osteological constraints of movement, it is equally important to recognize the possible constraints imposed by soft tissue structures such as the zygapophyseal capsule, interspinous ligament, supraspinous ligament, intervertebral disc and cervical musculature. These soft tissues may render the bone-on-bone maximum displacement point an underestimation or overestimation, because of their ability to help stretch and compress the neck. These structures aid in stabilizing the vertebral column during movement, by establishing the limitations on where structures can move in relation to one another. Therefore, future studies could model and incorporate the contributions or constraints of soft tissue structures in addition to the osteological constraints.

Another potential factor constraining neck ROM was the point of rotation between the cervical vertebrae. In this study, we chose to place the point of rotation at the centre point between the centra, following previous plesiosaur neck mobility studies [15,18]. In *N. borealis*, the centra are amphicoelous [23], which results in two slightly concave surfaces meeting one another from the respective cervical vertebrae. Inside of this space the cartilaginous intervertebral disc would have functioned to allow for movement between the cervical vertebrae, and brace the vertebral column against compressive forces. Because of this, we determined this point to be the logical placement for the point of rotation. However, given that plesiosaurian necks may be naturally flexed in their resting state [15–18] then the point of rotation may change along the column. Previous studies have suggested that the point of rotation in the cervical vertebral column may be more anteriorly or posteriorly placed [26]. As a control, we assumed that the point of rotation in *N. borealis* was at the same location throughout the entire cervical vertebral column. A future study could test the different point of rotation locations (i.e. at the zygapophyses) and assess how this affects the ROM found through this study.

As mentioned in the description of *N. borealis*, several of the cervical vertebrae were damaged during excavation [23], which resulted in missing ROM measurements (data are accessible via Dryad). Although this is not a true osteological constraint, it resulted in a number of vertebrae (C7–C9) not being accurately manipulated to bone-on-bone contact. We did not attempt to reconstruct the missing structures (i.e. neural spines, zygapophyses and cervical ribs) to avoid estimation errors associated with assumptions about the missing morphology.

There were also a number of osteological constraints in the neck of *V. dumerilii* in the V3DM trial. In the lateral mobility profile, contact occurred between the zygapophyses and neural arches (figure 5), and it should be noted that no cervical ribs are present in *V. dumerilii*. In the dorsoventral mobility profiles, the main constraints on movement were the zygapophyseal surfaces during dorsal elevation and the hypophyses of the centra during ventral flexion (figure 5).

### 4.3. Regional neck movement in *Nichollssaura borealis*

The osteological constraints may also result in regional neck mobility differences along the cervical vertebral column. *Nichollssaura borealis* was preserved with a great deal of lateral flexion in the anterior cervical vertebrae (figure 1). Figure 3 qualitatively shows that the lateral direction of movement is greater than the dorsoventral. The PCVM trial shows a possible trend towards decreasing ROM posteriorly along the neck (see electronic supplementary material, figure S.4). Although this study did not directly assess regional differences in mobility in any specific direction, given what can be observed in figure 1 and what the PCVM trial shows, it would be an informative line of questioning to explore. An analysis of how the changes in the cervical morphology relate to the modelled mobility could help quantify regional mobility in the neck. For now this work is outside the scope of this study, but may be a source of future study.

### 4.4. Ecological implications

Neck elongation evolved early on in Plesiosauria, and in other early sauropterygians [1,2]. Interestingly, the elongate neck was maintained, reduced and even regained, respectively, in a number of plesiosaurian groups over a 135 Myr period during the Mesozoic [1–4,7–12,15–18]. Since the function and adaptive value of the neck has remained unclear, because of a lack of modern analogues for comparison [15,17,18], it was a goal of this study to try and shed light on how the plesiosaur neck functioned. If the elongate neck remained in plesiosaurs for the entirety of their evolutionary history then it may have conveyed an adaptive advantage, perhaps for feeding [17,34]. However, although feeding with an elongate neck may have been advantageous, this may not be the driving force behind its initial evolution [35]. The elongate neck may have evolved in earlier plesiosaur lineages for another reason (e.g. developmental constraints or as novel hydrodynamic adaptation) and was later co-opted for feeding.

This study, like previous studies, quantified the function of the elongate neck [15–18]. Work by Zarnik [15] and Evans [17] showed the neck to be more laterally mobile than dorsoventrally. Evans [17] suggested that this was to facilitate a horizontal feeding pattern. Zammit *et al*. [18] found in elasmosaurs that the neck was more dorsoventrally mobile, when compared with lateral mobility. These differences could be the result of the angulation of the articular facets of the cervical vertebrae of the various groups [17]. For example, if the zygapophyses are more vertically oriented then dorsoventral mobility may be more prevalent. If the zygapophyses are more horizontally oriented then there may be more lateral mobility.

This study found that *N. borealis* possessed a neck that was most mobile in the lateral direction. These findings support what Evans [14] found in the plesiosaurs he studied (*C. eurymerus* and *M. leedsii*). *N. borealis* would have had a relatively large medio-lateral feeding envelope, which may have facilitated feeding in or along the substrate of the seafloor [13,17]. With wide sweeping motions *N. borealis* may have searched the seafloor for invertebrates and fish; however, no gut contents representing prey are known from the specimen. Prey gut contents from other plesiosaurs have previously contained invertebrates that potentially lived in the substrate of the seafloor [13]. Both this study and Evans's study [17] have shown that preferences for lateral neck movement may be common in some plesiosaurs and may represent a specific feeding regime.

As in other reptiles [36,37], *N. borealis* may have been capable of more complicated neck movements, such as retraction (i.e. an ‘S-shaped’ pose), rotation/torsion or even a combination of those. Snively *et al*. [26] showed that 3D modelling can be used to demonstrate these more complex movement patterns in extinct animals. In figure 1, the neck of *N. borealis* is preserved with a ‘U’ curve in the anterior most cervical vertebrae. This shows that neck retraction could have been a possible movement pattern in life. Although this study did not directly test that motion, the methodology presented in this study could be used for such an analysis. If retraction of the neck was possible, it could support suggestions that plesiosaurs were ambush predators that used lateral movements to retract the neck before striking for prey.

Feeding facilitated by lateral neck movement may have been confined to the smaller bodied plesiosaurs [15,17]. Zammit *et al*. [18] demonstrated that the larger elasmosaurs had greater dorsoventral movement, potentially representing a different feeding regime. Considering the range of body sizes and neck lengths found among plesiosaurs, it is reasonable to assume that the group had a range of ecologies. Particularly because the neck allows the head to interact with the environment, it is reasonable to assume that different movement patterns are present across plesiosaurs and that this will reflect ecological specializations. Therefore, understanding how the necks moved across larger samples of plesiosaurs may reveal a variety of functional capabilities that could further inform interpretations of plesiosaur ecology.

### 4.5. Summary and future directions

This study aimed to demonstrate how the plesiosaur neck functioned by assessing its ROM. The mobility profiles presented here reflect biologically realistic movement patterns of the neck of *N. borealis* because of the agreement between the *V. dumerilii* datasets (V3DM and RMV trials). We established the maximum and minimum ROM, which allowed us to bracket the possible ROM of *N. borealis* neck given a variety of assumptions. These assumptions included differences in intervertebral spacing that could result from compression or expansion of cartilaginous components of the neck. They also included what factors may have constrained the neck movement. This work showed that 3D modelling from CT scans is a viable approach for studying the mobility of the neck in the plesiosaur *N. borealis*.

The approach presented here could be applied to other plesiosaur specimens, such as the hyper-elongate-necked elasmosaurids. Differences in mobility patterns between groups of plesiosaurs may allow for inferences of ecological niche specializations, and why the elongate neck persisted across the group for as long as it did. Ultimately, an increased sample size of plesiosaur neck mobility will allow further understanding of the ecological and evolutionary history of the group.

## Supplementary Material

Supplementary Information 1

## Supplementary Material

Supplementary Information 2

## Data accessibility

Our data are deposited at Dryad: http://dx.doi.org/10.5061/dryad.pm7p6 [38].
