# Supplementary material for: A method for deducing neck mobility in plesiosaurs, using the exceptionally preserved Nichollssaura borealis
Source: R Soc Open Sci. 2018 Aug 1;5(8):172307. doi: 10.1098/rsos.172307 (PMC6124041; doi:10.1098/rsos.172307)
Supplement: Supplementary Information 2 [file rsos172307supp2.docx]

A

C

B


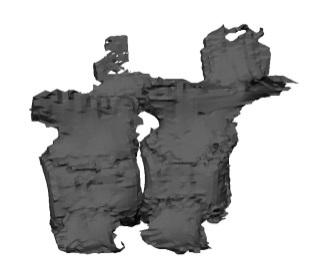

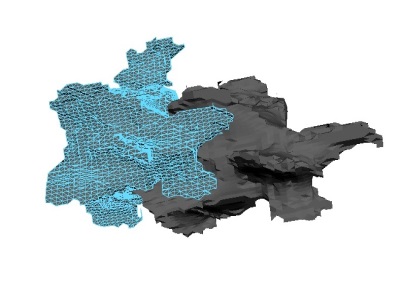

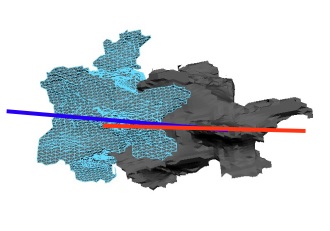

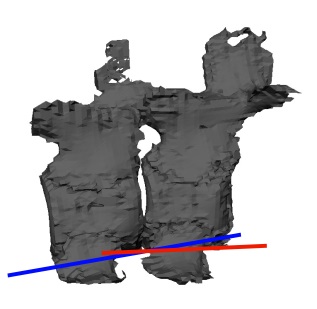

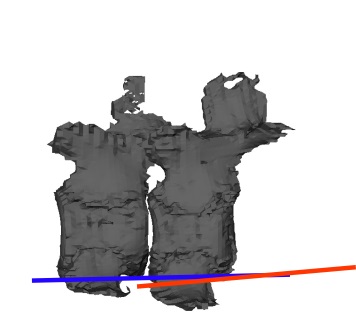

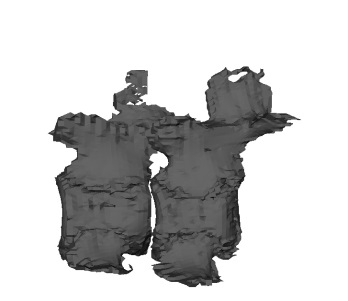


Figure S.1

MISM trial: Minimum intervertebral spacing articulation: C4 and C5 shown. **A)** Dorsal mp, and measurement rlv. **B)** Ventral mp, and measurement rlv. **C)** Lateral mp, and measurement dorsal view.


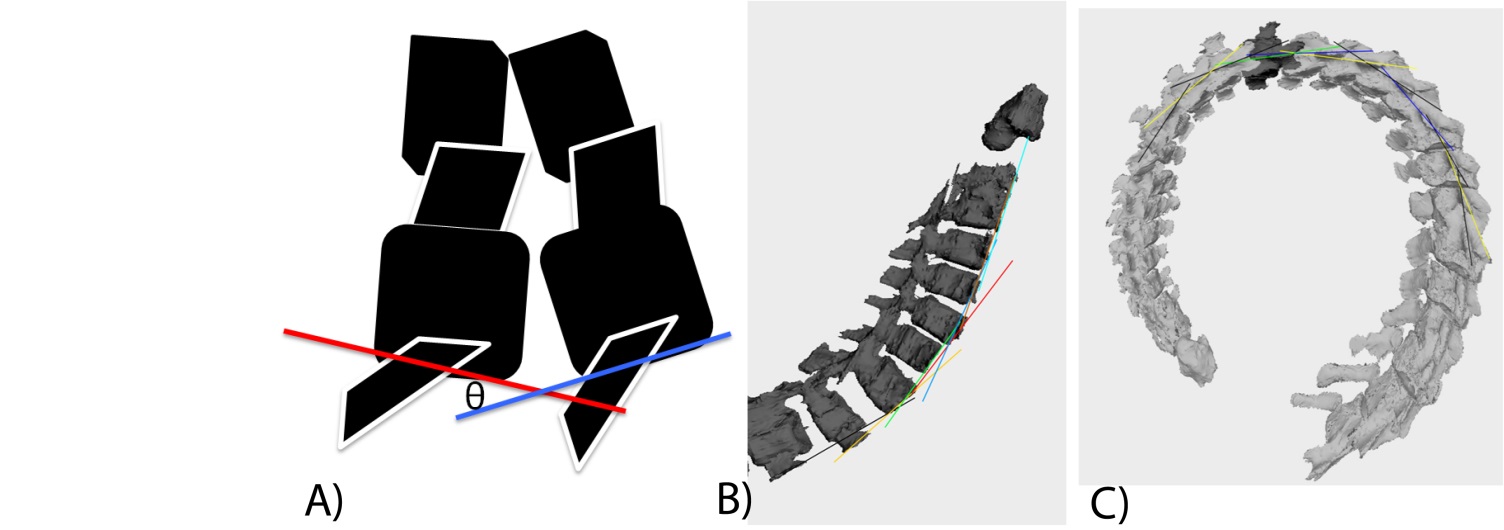


Figure S.2

**A)** Diagram of two cervical vertebrae in right lateral view. A straight line has been placed on the ventral margins of each of the centrum, one blue, and one red. Theta shows the angle measured for the intervertebral range of motion. **B)** Shows a series of intersecting lines placed for measurement on the Autodesk Maya model of *N. borealis*. **C)** Shows the placement of intersecting lines along the dorsal midline of the vertebrae of the Autodesk Maya model of *N. borealis* for the lateral range of motion measurements.


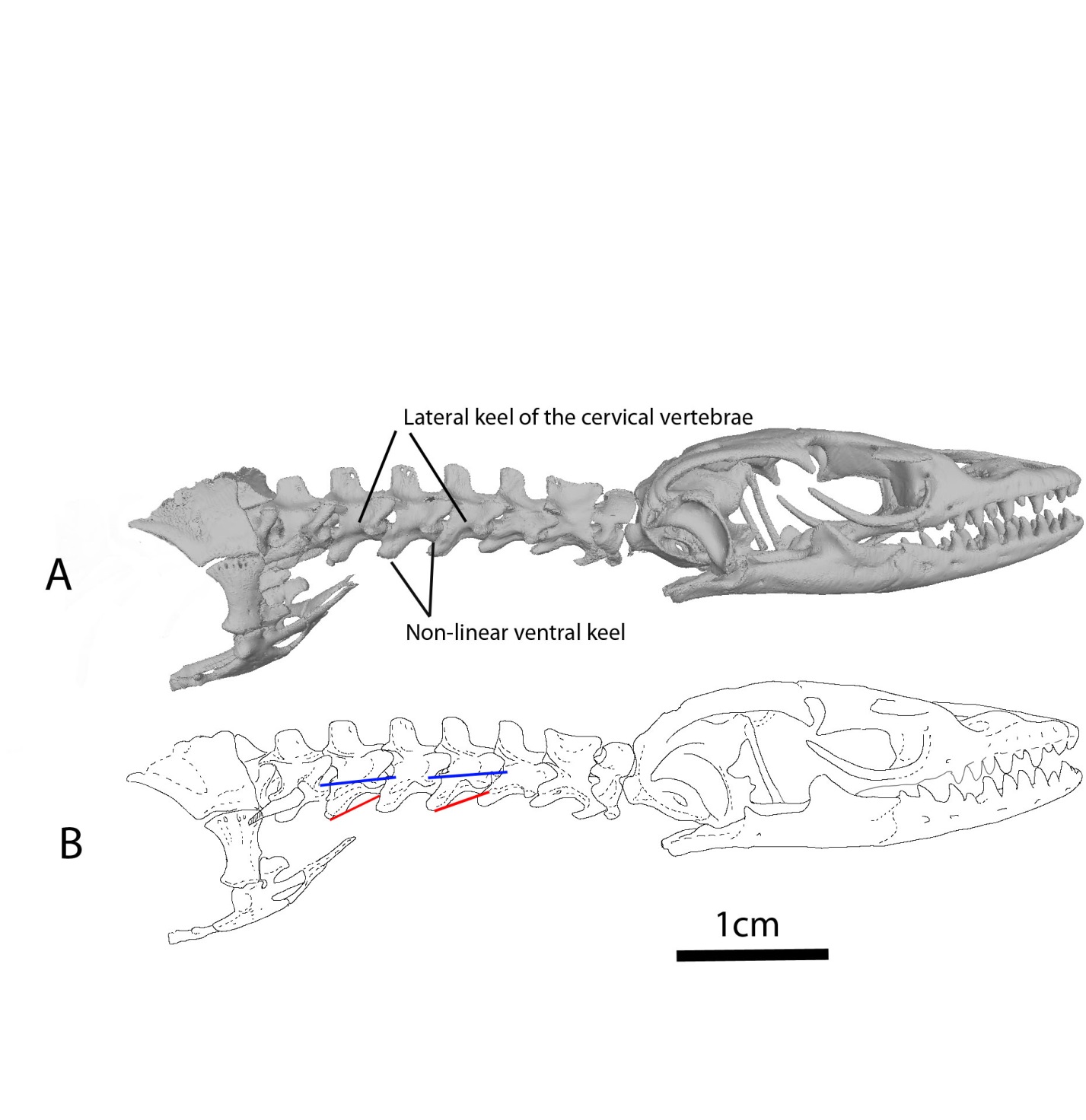


Figure S.3

*Varanus dumerilii* in right lateral view, shown both as a CT scan (A) and as a line drawing (B). **A)** Shows the location of the lateral keel and the non-linear ventral keel of the cervical vertebrae. **B)** Shows the blue line represents the series of lines (though no complete here) that were placed along the lateral keel for range of motion data collection. The red line placed along the ventral keel shows the non-linear nature of the ventral keel, and the primary reason why it was not used as the location for range of motion data collection.

**Figure S.4**

Range of motion (in degrees) is plotted against cervical vertebral manipulations for PCVM trial: paired cervical vertebral mobility. Lateral mobility is shown in blue, dorsal in orange, and ventral in grey.

Figure S.5

Range of motion (in degrees) is plotted against cervical vertebral manipulations for MISM trial: minimum intervertebral space mobility. Lateral mobility is shown in blue, dorsal in orange, and ventral in grey.
